# Hyperthermostable *Thermotoga maritima* xylanase XYN10B shows high activity at high temperatures in the presence of biomass-dissolving hydrophilic ionic liquids

**DOI:** 10.1007/s00792-016-0841-y

**Published:** 2016-05-30

**Authors:** Tianyi Yu, Sasikala Anbarasan, Yawei Wang, Kübra Telli, Aşkın Sevinç Aslan, Zhengding Su, Yin Zhou, Li Zhang, Piia Iivonen, Sami Havukainen, Tero Mentunen, Michael Hummel, Herbert Sixta, Baris Binay, Ossi Turunen, Hairong Xiong

**Affiliations:** South-Central University for Nationalities, College of Life Science, Wuhan, 430074 China; Department of Biotechnology and Chemical Technology, School of Chemical Technology, Aalto University, 00076 Aalto, Finland; Hubei University of Technology, Wuhan, 430068 China; Wuhan Sunhy Biology Co., Ltd, Wuhan, 430074 China; Department of Forest Products Technology, School of Chemical Technology, Aalto University, 00076 Aalto, Finland; Department of Bioengineering, Gebze Technical University, 41400 Gebze Kocaeli, Turkey

**Keywords:** GH10 xylanase, *Thermotoga maritima*, Extreme stability, Ionic liquids, Competitive inhibition, Expression in *Pichia pastoris*

## Abstract

The gene of *Thermotoga maritima* GH10 xylanase (TmXYN10B) was synthesised to study the extreme limits of this hyperthermostable enzyme at high temperatures in the presence of biomass-dissolving hydrophilic ionic liquids (ILs). TmXYN10B expressed from *Pichia pastoris* showed maximal activity at 100 °C and retained 92 % of maximal activity at 105 °C in a 30-min assay. Although the temperature optimum of activity was lowered by 1-ethyl-3-methylimidazolium acetate ([EMIM]OAc), TmXYN10B retained partial activity in 15–35 % hydrophilic ILs, even at 75–90 °C. TmXYN10B retained over 80 % of its activity at 90 °C in 15 % [EMIM]OAc and 15–25 % 1-ethyl-3-methylimidazolium dimethylphosphate ([EMIM]DMP) during 22-h reactions. [EMIM]OAc may rigidify the enzyme and lower *V*_max_. However, only minor changes in kinetic parameter *K*_m_ showed that competitive inhibition by [EMIM]OAc of TmXYN10B is minimal. In conclusion, when extended enzymatic reactions under extreme conditions are required, TmXYN10B shows extraordinary potential.

**Electronic supplementary material:**

The online version of this article (doi:10.1007/s00792-016-0841-y) contains supplementary material, which is available to authorized users.

## Introduction

Xylanases, members of glycosidases (O-glycoside hydrolases, EC 3.2.1.x), catalyse the endohydrolysis of 1,4-beta-D-xylosidic linkages in xylan (Collins et al. 2005). Thermostable xylanases are widely used in biotechnological processes that take place at high temperatures, such as those in the feed and pulp and paper industry (Vieille and Zeikus 2001; Kumar et al. 2016). Xylanases are also used in industrial applications for the deconstruction of plant cell walls to facilitate biofuel production from lignocellulose. Lignocellulosic materials from agricultural and forestry waste can serve as key substrates for second-generation biofuels due to their low cost and abundance (Yeoman et al. 2010; Bhalla et al. 2013). Xylanases are used in the decomposition of the hemicellulosic components of the biomass.

The application of thermostable enzymes in industrial processes, which are carried out at high temperatures, have several advantages, including extended reaction times and also stability against denaturing conditions other than heat (Yeoman et al. 2010). Elevated temperatures lead to faster reactions, higher mass transfer rates, lower viscosity, increased solubility of reactants and products, lowered risk of contamination from mesophilic microbes and therefore improved hydrolysis performance. A temperature of 100 °C offers advantages for the enzymatic treatment of lignocellulose (Hämäläinen et al. 2015). Enzyme activity at these temperatures requires extreme stability and preferably a long half-life of the enzyme. Highly thermostable xylanases from diverse thermophilic microbes have been described (Canakci et al. 2012; Shrivastava et al. 2013). Synergy effects between different enzymes are important in lignocellulose hydrolysis (Hämäläinen et al. 2015; Malgas et al. 2015).

Different mutagenesis methods have been adopted to improve the thermostability of xylanases, and these have led to significant improvements in the thermostability (Palackal et al. 2004; Xiong et al. 2004; Dumon et al. 2008; Wang et al. 2012; Li et al. 2013; Song et al. 2015). Even xylanases with low native thermostability can be engineered to have remarkably high stability levels (Jänis et al. 2001, 2004; Palackal et al. 2004; Xiong et al. 2004). Although the optimum temperature for the activity of *Dictyoglomus thermophilum* GH11 xylanase is around 85–90 °C, a mutant was generated with a slightly higher (about 5 °C) temperature optimum (Li et al. 2013), showing that protein engineering can improve the thermostability of hyperthermostable enzymes. With regard to hyperthermostable xylanases, those found in natural microbes still have the highest level of thermostability. The temperature optimum of *Thermotoga maritima* GH10 xylanase was 105 °C in a 5-min assay (Winterhalter and Liebl 1995; Reeves et al. 2000; Ihsanawati et al. 2005) and that of *Pyrodictium abyssi* GH10 xylanase was 110 °C (Andrade et al. 2001).

Hydrophilic ionic liquids (ILs) have been used successfully as dissolving agents in the pretreatment of lignocellulose for improved enzyme hydrolysis (Wahlström and Suurnäkki 2015). [EMIM]OAc is a common IL used in lignocellulose-dissolving experiments. These biomass-dissolving ILs are usually harmful to an enzyme, causing both unfolding of the structure and competitive inhibition of the enzyme activity (Vancov et al. 2012; Jaeger and Pfaendtner 2013; Li et al. 2013; Chawachart et al. 2014). The competitive inhibition of enzyme activity and the disruption of enzyme structure by [EMIM]-based ionic liquids has been observed also in molecular dynamics simulations (Jaeger and Pfaendtner 2013; Jaeger et al. 2015). Finding or engineering enzymes compatible with biomass-dissolving ILs would make it possible to study the activity of enzymes in lignocellulose hydrolysis or modification, together with high concentrations of biomass-dissolving ILs. In addition, high temperature and biomass-dissolving ILs form a model for extreme conditions to study the enzyme behaviour. In the present study, the *T. maritima* GH10 xylanase (TmXYN10B) was expressed in *Escherichia coli* and *Pichia pastoris.* The effect of the expression system on its enzymatic properties and the limits of *T. maritima* GH10 xylanase at high temperatures in the presence of biomass-dissolving ILs were then investigated.

## Materials and methods

### Strains, vectors and materials

*Escherichia coli* BL21 (DE3) (Transgen, China) was used as the host for prokaryotic protein expression. It was grown aerobically in Luria–Bertani broth with 100 µg of ampicillin/mL. The vector pET-22b(+) and shuttle vector pPIC9 (Invitrogen, China) were used for secretion of target proteins. All enzymes were purchased from Takara (Japan). Primers and genes were synthesised by Tsingke Biotech (China). *P. pastoris* GS115 (Invitrogen, Beijing, China) was used as the host for eukaryotic protein expression. The culture media were: minimal dextrose (MD), yeast extract peptone dextrose (YPD), buffered glycerol-complex (BMGY) and buffered methanol-complex (BMMY). All chemical reagents were of analytical pure grade.

### Construction of the recombinant plasmid

According to the reported complete genome of *T. maritima* MSB8 [NCBI (The National Center for Biotechnology Information) database reference sequence: NC_023151, 1,869,644 bp], the xylanase 10B gene in the 873531…874574 (Gene ID: 18092741, 1044 bp) region was selected to construct the TmXYN10B gene. The synthesised sequence was deposited in NCBI database (Gene ID: KR078269, 984 bp; protein ID: AKT33673, 328 amino acids). The TmXYN10B sequence was designed to avoid the *EcoR* I and *Bgl* II recognition sites in the construction of GS115-*Xyn10B* plasmid for expression in *P. pastoris*. The xylanase TmXYN10B sequence was optimised by removing rare codons and optimising the codon usage for expression in *P.pastoris.* The full-length xylanase gene TmXYN10B was synthesised by Tsingke Biotech. The mature gene was inserted into endonuclease sites of *Xho* I and *Nco* I in plasmid pET-22b(+). While the gene was inserted in pET-22b(+), the termination codon was removed to link the His-tag with the C-terminal of the TmXYN10B protein. Meanwhile, the full-length gene was inserted into the restriction endonucleases sites of *EcoR* I and *Not* I. The secretion signal sequence was from the expression vector. The two recombinant plasmids were preserved in *E. coli* DH5α.

### Expression of xylanase in *E. coli* and *P. pastoris*

The expression plasmid pET-22b(+)-*Xyn10B* was transformed into competent *E. coli* BL21 (DE3) cells. The cells were cultivated at 37 °C for 12 h. The protein expression was then induced by isopropyl-β-D-thiogalactopyranoside (IPTG) at a final concentration of 0.5 mM and incubated at 28 °C for 16 h. The pPIC9-*Xyn10B* plasmid was digested with *Bgl* II and then transformed into competent *P. pastoris* GS115 cells by electroporation. Transformants were screened on MD plates lacking histidine. Positive colonies were transferred into 5 mL of BMGY medium and grown at 30 °C for 2 days. The cells were pelleted by centrifugation and resuspended in 1 mL of BMMY containing 0.5 % methanol for 72 h to induce protein expression. Batch cultivation was carried out in a 20-L bioreactor (Sartorius, Germany) to grow the GS115-*Xyn10B* for producing the TmXYN10B protein. The cultivation parameters were as follows: agitation at 200 rpm (tip speed 0.6 m/s, two Rushton type impellers), aeration of 0.3 vvm (volume air per volume liquid per minute) and cultivation for 8 days at 28 °C (Xiong et al. 2009).

### Purification of the TmXYN10B protein

The culture supernatant of *E.coli* pET-22b-*Xyn10B* was collected by centrifugation at 4 °C and 10,000×*g* for 15 min. The cell-free supernatant was filtered with a 10-kDa membrane. The filtered supernatant was loaded to a 1 mL immobilised Ni^2+^ affinity column (Novagen, Wisconsin) and eluted with elution buffer (0.4 M imidazole, 0.5 M NaCl, and 20 mM Tris–HCl, pH 7.9). The eluent was concentrated and analysed by sodium dodecyl sulphate polyacrylamide gel electrophoresis (12 % SDS-PAGE). The protein concentrations were determined by the Bradford method, using bovine serum albumin (BSA) (Sigma-Aldrich, MO, USA) as the standard.

The culture supernatant of *P. pastoris* GS115 was collected by centrifugation for 15 min at 10,000×*g*, 4 °C. The cell-free supernatant was precipitated with ammonium sulphate (80 % saturation). After centrifugation (5000×*g* for 30 min, 4 °C), the pellet was dissolved in 25 % saturated ammonium sulphate in 20 mM phosphate buffer (pH 6.0). The *P. pastoris*-expressed enzyme (~5 μg) was deglycosylated by 3000 U of endo-β-N-acetylglucosaminidase H (Endo H) at 37 °C for 2 h, according to the manufacturer’s instructions (New England Biolabs, MA, USA). The deglycosylated and untreated enzymes were analysed by SDS–PAGE. According to manufacturer, the apparent molecular weight of endo H is 29 kDa. The molecular weight marker was PageRuler Prestained Protein Ladder, 10–180 kDa (ThermoFisher, Scientific, MA, USA).

### Enzyme activity assays

The enzymatic activities of the produced xylanases were determined by reaction in a 1 mL mixture containing 100 μL of appropriately diluted enzymes and 900 μL 0.5 % (w/v) beechwood xylan (X4252, Sigma-Aldrich, MO, USA), which was dissolved in 50 mM phosphate buffer, pH 6.0. The DNS method with a 10-min assay length was used for determination of the enzyme activity (Xiong et al. 2004). Enzyme assays (10 min) were also performed with up to 2.0 M sodium chloride at 90 °C, pH 5. The specific activity of TmXYN10B was measured at 90 °C and pH 5. One unit (IU) of enzyme activity was defined as the amount of enzyme releasing 1 micromole of reducing sugars per min. The half-life was determined by incubating the enzyme samples at 100 °C (pH 5, initially at room temperature) for various time intervals. Thereafter, the remaining activity was measured at 90 °C, pH 5, with a 10-min assay. All these experiments were done with the enzymes purified as described above unless otherwise stated. In the assays, 0.1 mg/mL of BSA was used as a stabiliser.

### Enzyme activity in the presence of ILs

The effect of 1-ethyl-3-methylimidazolium acetate ([EMIM]OAc) (BASF, Germany), with a purity of ≥95 %, 1-ethyl-3-methylimidazolium dimethylphosphate ([EMIM]DMP) (IoLiTec, Germany), with a purity of ≥98 %, and 1,5-diazabicyclo[4.3.0]non-5-enium acetate ([DBNH]OAc) on the temperature-dependent activity of TmXYN10B was investigated with the enzyme expressed in *P.pastoris.* [DBNH]OAc was prepared by slowly adding equimolar amounts of acetic acid (glacial, 100 %, Merck, Germany) to 1,5-Diazabicyclo[4.3.0]non-5-ene (99 %, Fluorochem, UK), while diverting the enthalpy of the exothermic reaction by active cooling. The IL experiments were done with the non-purified supernatant enzyme (precipitates were removed) produced from *Pichia*. The amount of total supernatant protein including the enzyme in one experiment was below 0.1 mg/mL. 0.1 mg/mL of BSA was used as a stabiliser. The experiments were done at pH 7 in 50 mM citrate–phosphate buffer using 1 % birchwood xylan (Sisco Research Laboratories, India) as a substrate. The activity at 105 °C was measured in closed tubes incubated in a silicon oil bath. The time-dependent activity of TmXYN10B with [EMIM]OAc was measured at 75 and 90 °C, pH 7. A pH of 7 was used to avoid precipitation, which easily occurs at a lower pH. The reaction products were measured by a DNS assay. The pH was adjusted each time after the addition of the IL to the reaction solution. Xylose standards with IL were used to determine the amount of the produced sugars in the presence of ILs.

The kinetic parameters with and without [EMIM]OAc were determined by a DNS assay, with 30-min incubations at pH 7 and using 1 % birchwood xylan as the substrate. The kinetic parameters were calculated by hyperbolic regression analysis in Hyper32 software (http://homepage.ntlworld.com/john.easterby/software.html). The relative *V*_max_ was calculated to illustrate the effect of [EMIM]OAc on the kinetics values.

### Molecular docking by SwissDock

Molecular docking of [EMIM]^+^ and [DBNH]^+^ cations by SwissDock (http://www.swissdock.ch/) to the TmXYN10B structure (PDB code 1VBR) was done in the same way as described earlier for *T. aurantiacus* GH10 xylanase (Chawachart et al. 2014). The cation structures were energy minimised by MM2 in ChemBio3D Ultra 12.0 (CambridgeSoft, UK) for docking experiments. The accurate mode option of SwissDock was used, and flexibility was allowed for the ligand.

## Results

### Cloning and expression of the TmXYN10B gene

**Fig. 1:**
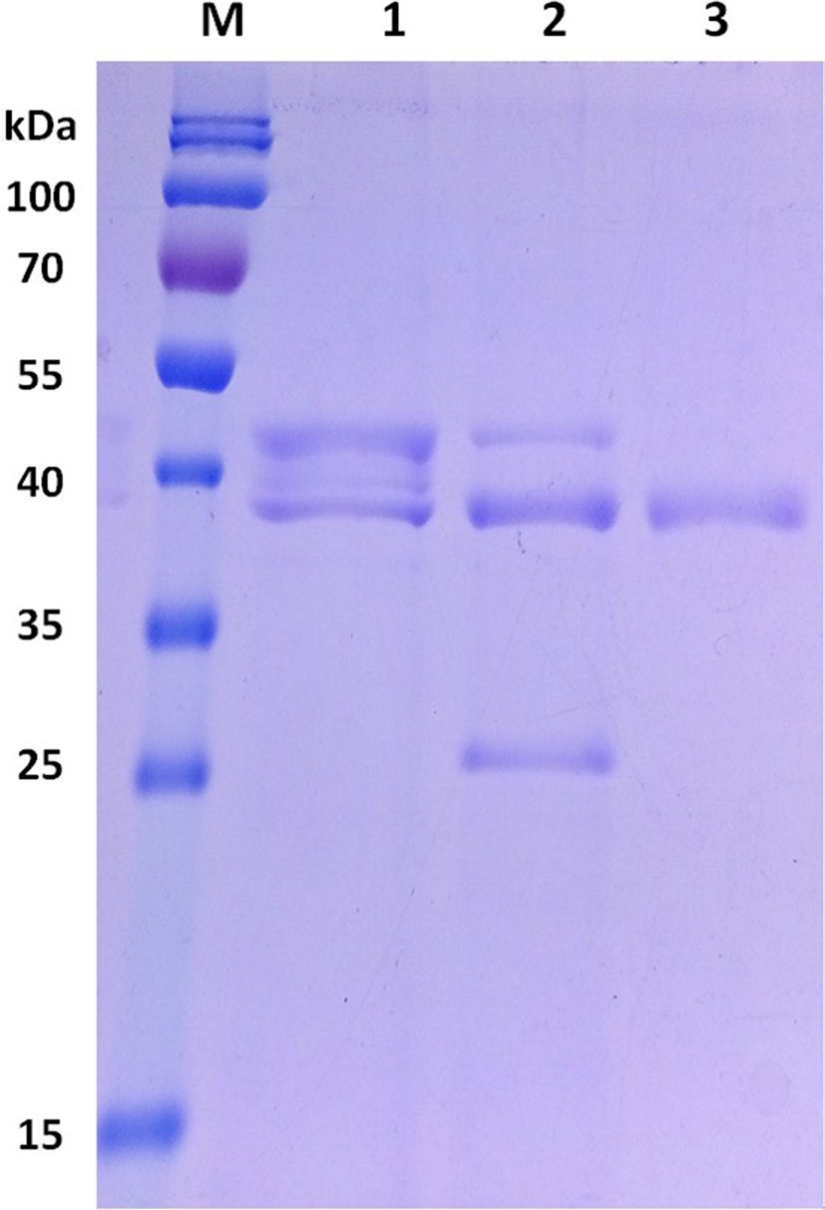
SDS-PAGE analysis of TmXYN10B proteins. *M* PageRuler Prestained Protein Ladder, 10–180 kDa. *1* the recombinant TmXYN10B expressed in *P. pastoris*; *2* TmXYN10B after deglycosylation with endo H (the lower band is the 29 kDa endo H); *3* TmXYN10B expressed in *E. coli*. About 5 μg of protein was loaded into each sample lane

The xylanase TmXYN10B sequence was obtained from the complete genome of *T. maritima* MSB8. The protein structure is known (PDB code 1VBR, 324 amino acids). After optimising the codon usage of the gene, the synthesised TmXYN10B gene (GenBank: KR078269) was constructed and expressed in *E. coli* and *P. pastoris*. The prokaryotic expression of TmXYN10B was induced by IPTG in *E. coli* BL21 (DE3) under the control of the T7 promoter, and the eukaryotic expression was induced by methanol in *P. pastoris* GS115 under the control of the AOX promoter. The TmXYN10B proteins were produced in the culture medium, from which they were purified and identified in SDS-PAGE. Protein sizes close to 40 kDa, consistent with the predicted size, were produced in *E. coli* and *P. pastoris* (Fig. 1). The protein produced in *E. coli* had a size of 38 kDa, and the protein produced in *P. pastoris* had a size of 42 kDa, indicating that TmXYN10B produced in *P. pastoris* was glycosylated. After treatment with Endo H specific for N-glycosylation, a second protein version appeared. This protein had an apparent molecular mass of 38 kDa, which was the same as that of the enzyme produced in *E. coli* (Fig. 1). The amino acid sequence of TmXYN10B contains several theoretical N-glycosylation sites (not shown).

### Enzymatic properties of TmXYN10B

**Fig. 2:**
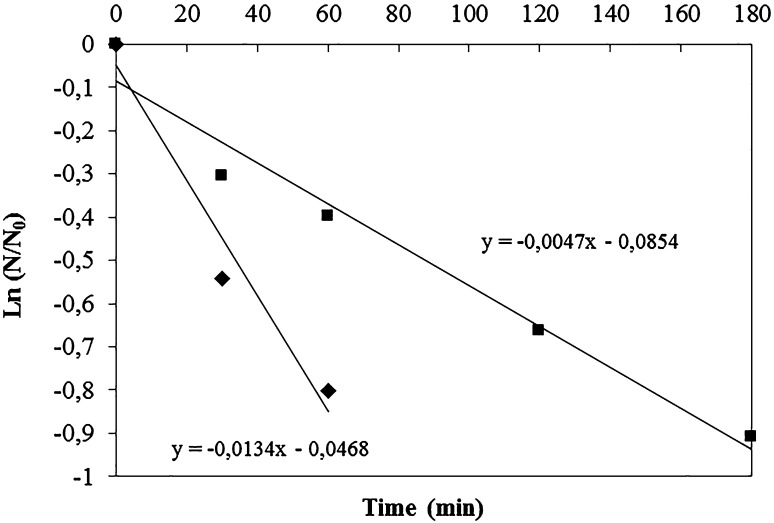
Inactivation of TmXYN10B at 100 °C (pH 5). *E. coli*-expressed TmXYN10B (*filled rhombus*) and *P. pastoris*-expressed TmXYN10B (*filled square*). In *Y*-axis, *N*_0_ is the activity value at time point 0 and *N* at each incubation time point. The *figure* shows the inactivation graph of *E. coli*-expressed enzyme for the initial 0–60 min (half-life 48 min) and for 0–180 min (half-life 130 min) in *P. pastoris*-expressed enzyme (see Fig. S1 in Supplemental Data)

The enzyme inactivation of TmXYN10B expressed in *E. coli* and *P.pastoris* as a function of time was compared (Fig. 2; see also Supplemental Data Fig. S1). When incubated at 100 °C and pH 5, the half-life times of TmXYN10B expressed in *E. coli* and *P.pastoris* were about 48 and 130 min, respectively (Fig. 2 and Fig. S1 in Supplemental Data). Therefore, the thermostability assay showed that the enzyme produced in *P.pastoris* was more stable than the enzyme produced in *E. coli*. Although the underlying reason was not studied, it could be due to the difference in glycosylation (from *Pichia*) or His-tag (in *E. coli* expression). The enzyme produced in *P.pastoris* had a wider pH stability region on both the acidic and alkaline side (not shown). The results are in line with the earlier studies showing that the expression systems can have a significant effect on the activity and stability of enzymes (Anbarasan et al. 2010).

At 90 °C and pH 5, the specific activity of TmXYN10B expressed in *E. coli* was 375 IU/mg, whereas the specific activity of TmXYN10B expressed in *P.pastoris* was 110 IU/mg. The lower activity of the enzyme expressed from *Pichia* may be caused by lower purity. However, given the lack of significant other impurities (Fig. 1), the lower bands in *Pichia*-expressed protein (Fig. 1, lane 1) likely represent a glycosylation variant and a non-glycosylated form. Glycosylation may also have decreased the specific activity.

**Fig. 3:**
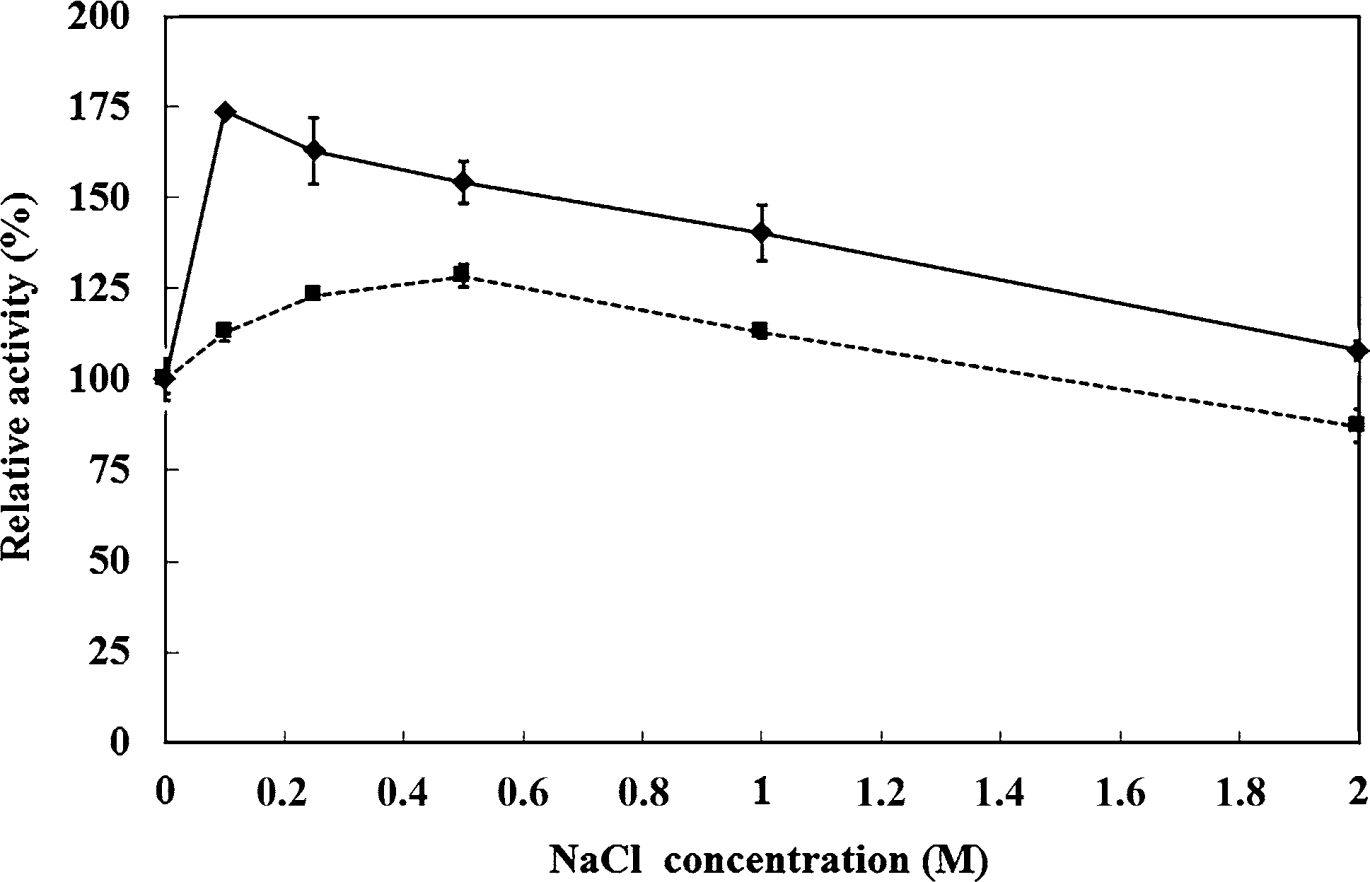
Effect of NaCl concentration on the activity of TmXYN10B. The enzyme expressed in *E. coli* (*filled rhombus*) and *P. pastoris* (*filled square*). The enzyme reaction was done at 90 °C, pH 5, for 10 min

The effect of sodium chloride on the activity of TmXYN10B produced in *E.coli* and *P.pastoris* is shown in Fig. 3. TmXYN10B was clearly activated by sodium chloride, as shown in earlier studies (Winterhalter and Liebl 1995). However, in the present study, the activation of the TmXYN10B enzyme in response to sodium chloride differed in *E. coli* and *P.pastoris*, with significantly higher activation when expressed in *E. coli*, with the C-terminal His-tag and without glycosylation. For both enzyme forms, the activity in 2 M NaCl was close to same.

### Thermal activity of TmXYN10B in the presence of ILs

**Fig. 4:**
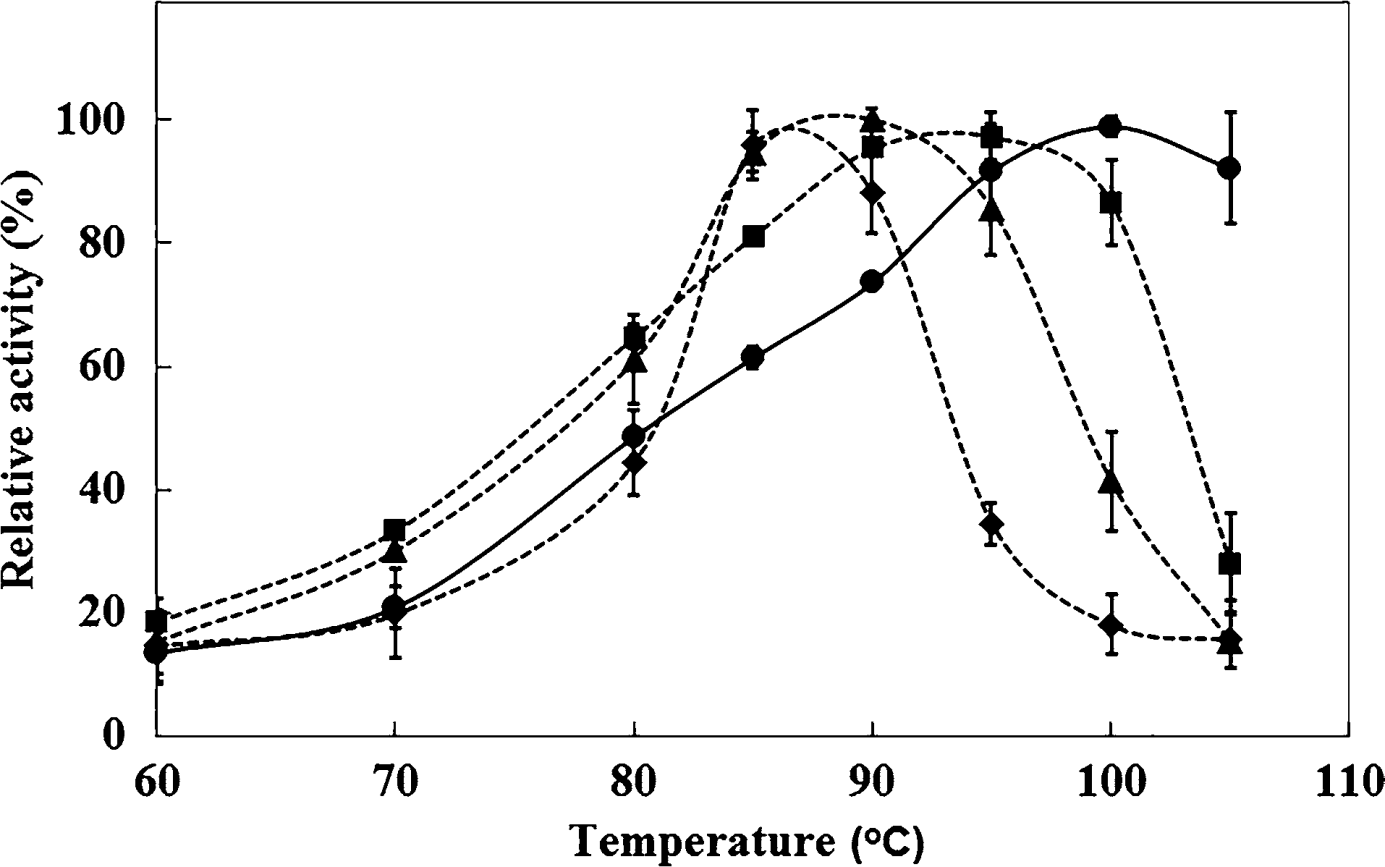
Effect of [EMIM]OAc on temperature-dependent activity of *P. pastoris*-expressed TmXYN10B. The experiments were done at pH 7, with 30-min incubation. The maximal activity in each IL concentration was defined as 100 %. *Symbols*: without [EMIM]OAc (*filled circle*) and with 15 % (*filled square*), 25 % (*filled triangle*), and 35 % (*filled rhombus*) [EMIM]OAc

The ability of highly thermophilic enzymes to tolerate ILs was tested earlier. The results showed that [EMIM]OAc decreased significantly the apparent temperature optimum of *D. thermophilum* and *T. aurantiacus* xylanases and melting temperature (*T*_m_) of *T. aurantiacus* xylanase (Li et al. 2013; Chawachart et al. 2014). TmXYN10B is more thermostable, with a higher temperature optimum (10–25 °C higher) than other xylanases studied earlier. The decreases in the temperature optimum of TmXYN10B were 5, 10 and 15 °C, with 15, 25 and 35 % of [EMIM]OAc, respectively (Fig. 4). Thus, in 15–35 % aqueous solutions, the extremophilic TmXYN10B still behaved as a highly thermophilic enzyme.

**Fig. 5:**
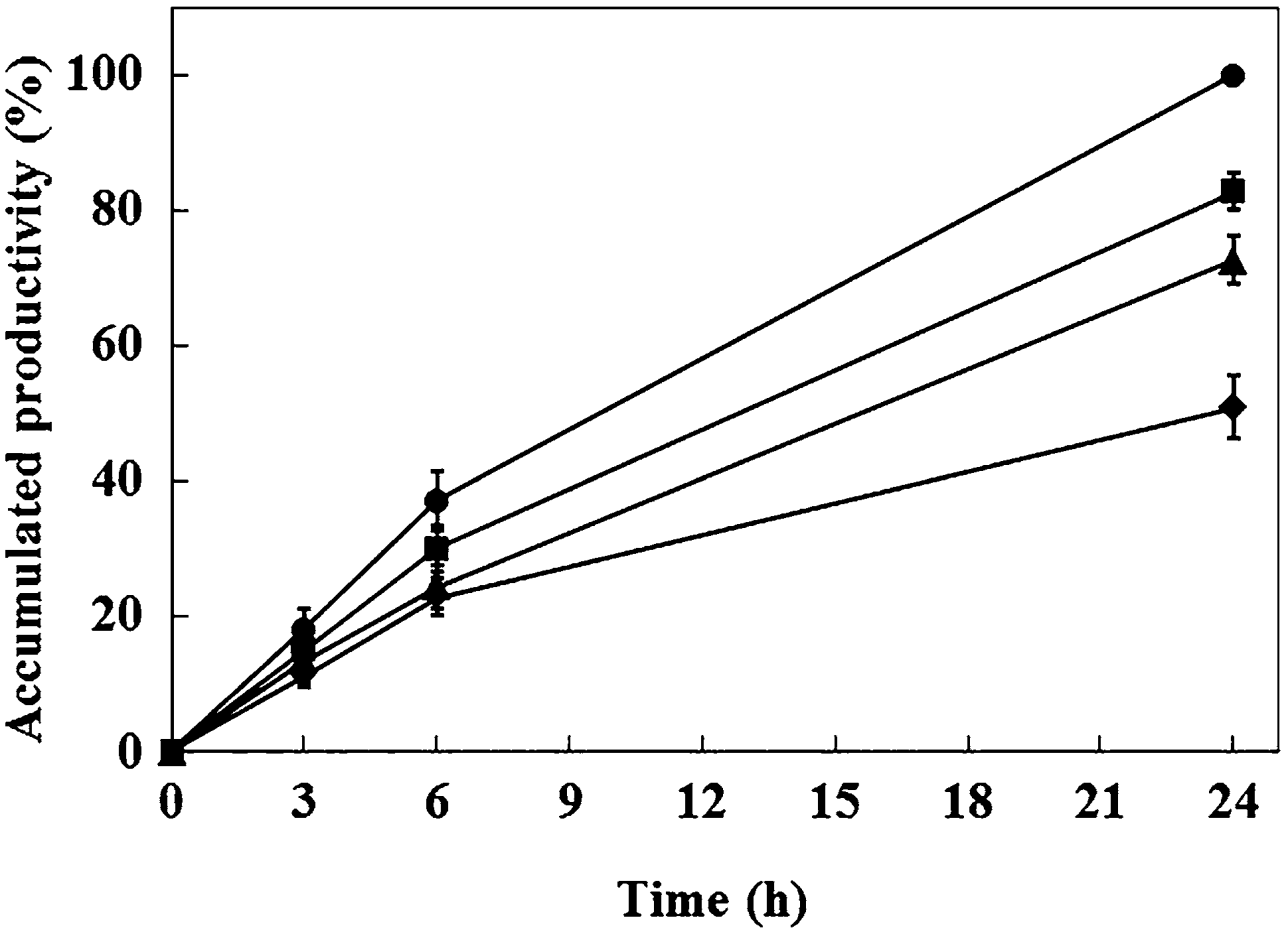
Time-dependent activity of TmXYN10B with [EMIM]OAc. The reaction was performed with 1 % birchwood xylan at 75 °C, pH 7. *Symbols*: without [EMIM]OAc (*filled circle*) and with 15 % (*filled square*), 25 % (*filled triangle*), and 35 % (*filled rhombus*) [EMIM]OAc. Relative activity (%) in the *X*-axis shows the relative production of the cleavage products from the same enzyme amount in the different assays

**Fig. 6:**
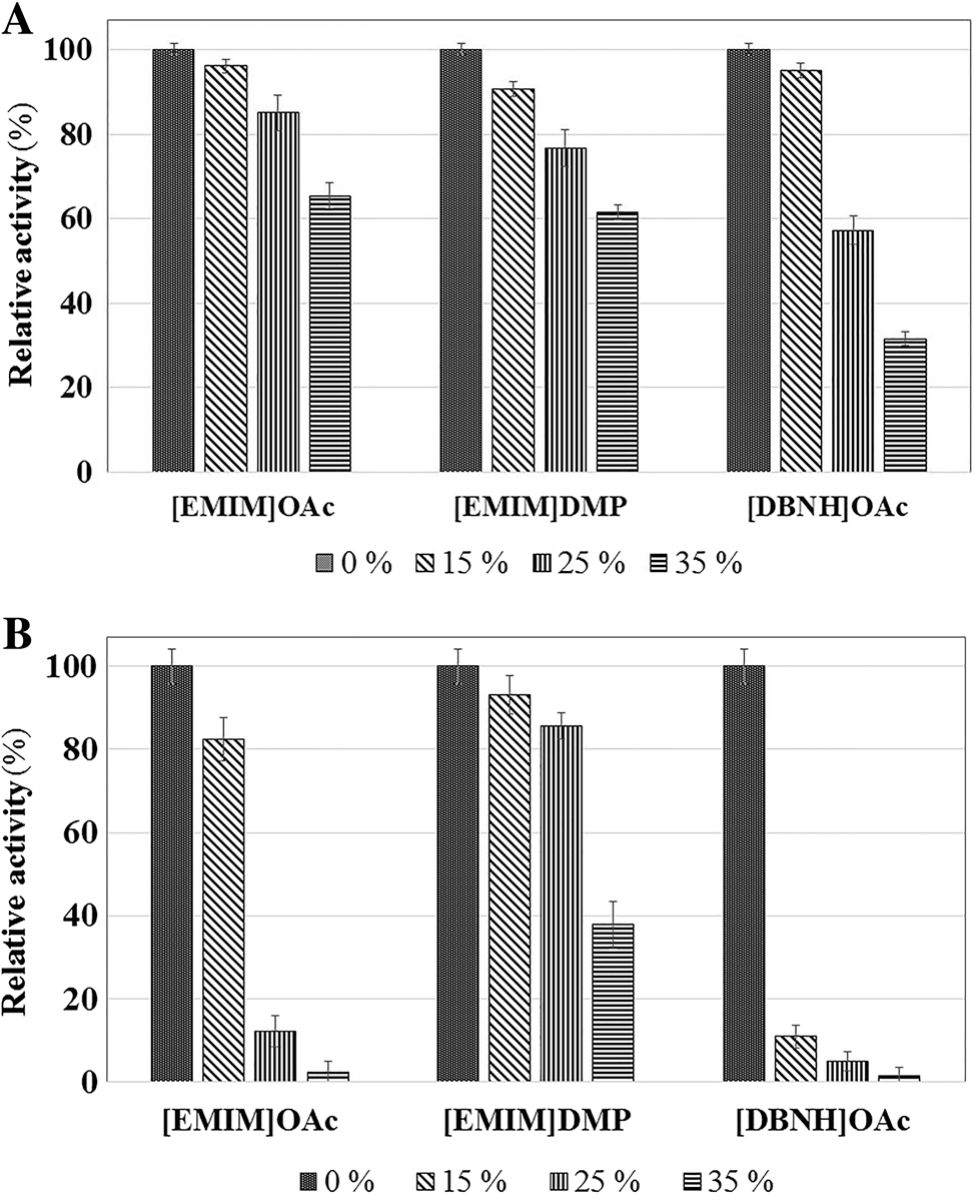
Activity of TmXYN10B in ILs at 90 °C. The reactions were performed at pH 7 with 1 % birchwood xylan in **a** 30-min and **b** 22-h incubations

With regards to the activity as a function of time, at 75 °C, the enzyme remained active during incubation for 24 h in 15 and 25 % [EMIM]OAc. Although the graphs bend, they still show that the enzyme was highly active during the whole time period. The ILs lowered the enzyme activity, but some activity remained even in 35 % [EMIM]OAc (Fig. 5). High stability in ILs was seen even at 90 °C, at which temperature we tested three ILs ([EMIM]OAc, [EMIM]DMP and [DBNH]OAc), and the results are shown for 30-min and 22-h incubations (Fig. 6a, b). All three ILs were tolerated quite well during incubation for 30 min, TmXYN10B showing the highest activity in [EMIM]OAc (Fig. 6a). The reaction product accumulation at 90 °C during 22 h showed close to the same level in 15 % [EMIM]OAc and 15 and 25 % [EMIM]DMP than without IL, whereas 25 and 35 % [EMIM]OAc, 35 % [EMIM]DMP and [DBNH]OAc decreased remarkably the enzyme activity (Fig. 6b). Surprisingly, now [EMIM]DMP was much better tolerated than [EMIM]OAc or [DBNH]OAc (Fig. 6b). These results pointed to significant differences in the effects of closely related ILs on the activity and stability of hyperthermostable TmXYN10B under differing conditions.

The kinetic parameters showed that [EMIM]OAc decreased *V*_max_ and the catalytic performance *V*_max_/*K*_m_ ~ 1.6 to 3.4-fold at 70–100 °C (Table 1; the corresponding graphs are shown in Supplemental Data Fig. S2). There was no dramatic increase in the kinetic parameter *K*_m_, reflecting substrate binding to enzyme, in response to [EMIM]OAc. In addition, the *K*_m_ increased only 1.8-fold from 0.69 to 1.22 mg/mL when the temperature was increased from 70 to 100 °C, indicating that the enzyme binds well to the substrate, even at 100 °C.

**Table 1:** Kinetic parameters of TmXYN10B

Without IL	*K*_m_ (mg/mL)	Relative *V*_max_	*V*_max_/*K*_m_
70 °C	0.69 ± 0.06	1.00 ± 0.07	1.4
90 °C	0.72 ± 0.11	2.47 ± 0.03	3.4
100 °C	1.22 ± 0.17	3.67 ± 0.05	3.0
With 15 % [EMIM]OAc			
70 °C	0.51 ± 0.04	0.47 ± 0.01	0.9
90 °C	1.41 ± 0.09	1.34 ± 0.01	1.0
100 °C	1.39 ± 0.20	1.57 ± 0.02	1.1

In relative *V*_max_, the value at 70 °C without [EMIM]OAc is defined as 1. The kinetic values are for the same amounts of enzyme. As TmXYN10B expressed in *P.* *pastoris* was not purified, only the relative *V*_max_ is reported

### Molecular docking of IL cations to the enzyme active site

**Fig. 7:**
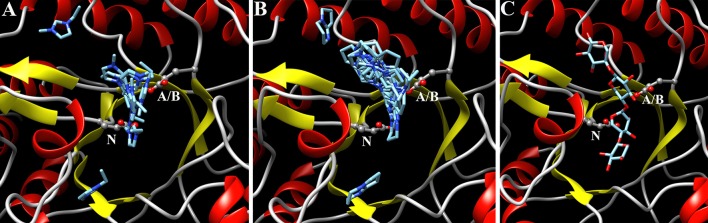
Molecular docking of IL cations and xylotetraose to the structure of TmXYN10B in SwissDock. The major binding positions are shown in the enzyme active site for the [EMIM]^+^ cations (**a**) and [DBNH]^+^ cations (**b**). The highest energy binding position of xylotetraose detected by SwissDock is shown in (**c**). The side chains of acid/base (A/B) and nucleophile (N) catalytic amino acids are shown

The molecular docking by SwissDock with the [EMIM]^+^ and [DBNH]^+^ cations was done in the same way as described earlier for *T. aurantiacus* GH10 xylanase (Chawachart et al. 2014). SwissDock grouped 256 docking poses to over 30 clusters. Ten clusters containing 80 poses were detected in the active site for [EMIM]^+^ cations, and 17 clusters containing 104 poses were detected in the active site for [DBNH]^+^ cations. Figure 7a and b show the first hits of every cluster bound to the active site. The highest binding energy for the [DBNH]^+^ cations was −6.54 kcal/mol, and it was −6.77 kcal/mol for the [EMIM]^+^ cations. The average binding energies (±standard deviation) of all docking poses in the active site were −6.18 ± 0.29 kcal/mol for the [EMIM]^+^ cations and −6.04 ± 0.30 kcal/mol for the [DBNH]^+^ cations. These differences in the binding energies are likely too small for having functional significance. The extent of the binding areas is probably more important. Figure 7c shows the location of the substrate (xylotetraose) binding site in the active site. The IL cations bound to the same areas, especially those close to catalytic nucleophile and acid/base catalysts.

When xylotetraose was docked to the active site, the highest binding energy was −10.51 kcal/mol for a structure in which the glycosidic oxygen and C1 of a xylose ring were close to an acid/base and nucleophile, respectively.

## Discussion

Hydrophilic biomass-dissolving ILs usually have harmful effects on enzymes. The thermostability and halophilicity of proteins appear to protect against such effects (Pottkämper et al. 2009; Zhang et al. 2011; Chawachart et al. 2014). Thermostability also seems to protect against structural unfolding by ILs (Chawachart et al. 2014). Both high halophilicity and thermophilicity are properties of TmXYN10B. Furthermore, factors other than high stability are also needed for elevated activity in ILs (Li et al. 2013; Chawachart et al. 2014). One such factor is tolerance to competitive inhibition caused by IL molecules. The present study indicates that *T. maritima* xylanase is highly tolerant to both enzyme inactivation and competitive inhibition of enzyme activity by ILs.

The temperature-dependent activity assays revealed a likely effect of [EMIM]OAc on the structure of TmXYN10B. [EMIM]OAc steepened the activity increase as a function of temperature as shown in Fig. 4. This was particularly clear in the comparison of the ratio of activities at 85 and 80 °C, which were much higher in 25 and 35 % [EMIM]OAc than without IL or in 15 % IL (Table 2). Accordingly, the Arrhenius activation increased with increasing IL concentration (Supplemental Data Fig. S3) This behaviour indicates that the TmXYN10B is more rigid in 25 and 35 % [EMIM]OAc, thus requiring higher amount of thermal energy for the enzyme functioning. The increase of viscosity in the reaction solution by [EMIM]OAc was probably not the reason for this effect, as was reported elsewhere that viscosity increases first in above 40 % [EMIM]OAc concentration (Quijada-Maldonado et al. 2012).

**Table 2:** Ratio between activities at 85 and 80 °C in [EMIM]OAc

[EMIM]OAc	0 %	15 %	25 %	35 %
85/80 °C	1.27	1.25	1.55	2.16

The values are based on activities in Fig. 4

While *T. aurantiacus* GH10 xylanase lost about 50 % of its activity at 60 °C in 25 % [EMIM]OAc (Chawachart et al. 2014), TmXYN10B lost only 15 % of its activity at 90 °C in 25 % [EMIM]OAc (30-min standard assays). Furthermore, as compared to the disulphide bridge mutant of *D. thermophilum* GH11 xylanase (Li et al. 2013), TmXYN10B also showed exceptional tolerance to [EMIM]OAc. With a temperature optimum of 95 °C the disulphide mutant of *D. thermophilum* GH11 xylanase lost over 80 % of its activity in 25 % [EMIM]OAc at 75 °C during 30 min of incubation, whereas TmXYN10B retained about 70 % of its activity at 75 °C during 24 h of incubation in 25 % [EMIM]OAc. *Volvariella volvacea* xylanase E2 was highly tolerant at the enzyme’s optimum temperature (not reported in the study) to [EMIM]OAc showing 14 % inactivation in 12-h assay (with pNP-xylose) (Thomas et al. 2011). However, the *Volvariella volvacea* enzymes have their optimum temperatures at 50–60 °C range (Li et al. 2005; Zheng et al. 2013). These comparisons show that TmXYN10B has unparalleled activity at temperatures close to boiling point in a biomass-dissolving ionic liquid, [EMIM]OAc.

We previously studied the effect of a high temperature on the behaviour and kinetic parameters of extremophilic xylanases (Li et al. 2015). In *D. thermophilum* GH11 xylanase, which has an apparent temperature optimum at 90 °C, the *K*_m_ value increased 5-fold in response to a shift in temperature from 90 to 100 °C. In contrast, with the disulphide mutant (temperature optimum at 95 °C), the *K*_m_ increased only 2-fold. In the present study, the *K*_m_ of TmXYN10B increased only 1.7-fold from 90 to 100 °C. These results show that at boiling point the substrate is binding well in those xylanases that have their temperature optimum at 95–100 °C.

Previous research showed that [EMIM]OAc increased the *K*_m_ of xylanases, with a 9.5-fold increase in the *K*_m_ of *D. thermophilum* GH11 xylanase in 15 % [EMIM]OAc and a 3.5-fold increase in the *K*_m_ of *Thermoascus aurantiacus* GH10 xylanase (Li et al. 2013; Chawachart et al. 2014). An increase in the *K*_m_ that is accompanied by only minor effects on the *V*_max_ indicates competitive inhibition. GH10 xylanase showed lower competitive inhibition than GH11 xylanase (Chawachart et al. 2014). For TmXYN10B, the increase in the *K*_m_ in 15 % [EMIM]OAc was minimal at 90–100 °C (2.0-fold and 1.1-fold, respectively) and absent at 70 °C (Table 1). These results show that the competitive inhibition is very low in TmXYN10B. However, *V*_max_ decreased approximately 2-fold by 15 % [EMIM]OAc showing activity inhibition by some other mechanism, e.g. by a rigidifying effect of ILs on TmXYN10B. Monosaccharides and oligosaccharides are known to inhibit cellulases, which can be a significant problem in the hydrolysis of lignocellulose (Collins et al. 2005). A study of extremophilic xylanase reported weaker binding of short sugar compounds at higher temperatures (Li et al. 2015). Therefore, when highly thermophilic xylanase enzymes are needed for biocatalytic processes under extreme conditions having also multiple inhibiting factors in the reaction mixture, the *T. maritima* GH10 xylanase appears to be a promising enzyme (Jia et al. 2012). TmXYN10B is able to resist the combined effect of more than one highly denaturing factor. The internal mobility of the proteins increases and degree of hydrogen bonding decreases with increasing temperature, and hydrogen bonds in the protein are replaced by hydrogen bonds to water (Cooper 2000). Hydrophilic ionic liquids also break hydrogen bonds and behave as surfactants denaturing enzymes (Zhao 2010).

A previous study reported that [EMIM]OAc showed stronger competition inhibition of GH11 than GH10 xylanases and that this was reflected in a larger binding area for [EMIM]^+^ cations in the active site of the GH10 xylanase, as detected by molecular docking (Chawachart et al. 2014). In the present study, molecular docking of the IL cations and xylotetraose showed that the IL cations occupied (probably transiently) the same positions as the substrate (Fig. 7). As shown by the results of the molecular docking analysis, while the [DBNH]^+^ cation docked to 17 different cluster positions in the active site, the [EMIM]^+^ cation docked to only 10 cluster positions. This may indicate that the slightly larger bicyclic structure of [DBNH]^+^ cations binds and fills the active site in a transient manner better than smaller monocyclic [EMIM]^+^ cations. Therefore, the inhibition of the enzyme is likely to be stronger, as was seen in the present experimental studies. The highest binding energy for the [EMIM]^+^ cations was −7.8 kcal/mol in *T. aurantiacus* XYN10A (−6.8 kcal/mol in TmXYN10B), whereas the binding energy for good xylotetraose positioning was −8.9 kcal/mol in *T. aurantiacus* (Chawachart et al. 2014). The higher binding energy for xylotetraose in the *T. maritima* enzyme (−10.5 kcal/mol) and possibly also the lower binding of the [EMIM]^+^ cations may explain why TmXYN10B was more tolerant to ILs than the *T. aurantiacus* enzyme. As the substrate has higher binding affinity in TmXYN10B, it is more efficient than in the *T. aurantiacus* enzyme at replacing the IL molecules from the active site.

Comparing the results obtained using the three ILs, the higher activity of TmXYN10B in [EMIM]OAc than [EMIM]DMP in short assays (30 min) confirmed the influence of anions (acetate vs dimethylphosphate) on the enzyme’s activity, although the difference was quite small. However, the activity order was different in long incubations (22 h), with [EMIM]DMP being best tolerated. This finding was likely due to dimethylphosphate being less denaturing than acetate. The comparison of the two ILs with acetate as the anion showed that [DBNH]^+^ cations had a much more inactivating effect than [EMIM]^+^ cations in long incubations. [DBNH] ^+^ cations also resulted in lower activity in short assays at higher IL concentrations. In conclusion, among the ILs tested, [EMIM]DMP showed the most promise during long incubations with TmXYN10B at very high temperatures, such as 90 °C.

While the pretreatment of lignocellulose with ILs improves subsequent enzymatic lignocellulose hydrolysis, the use of enzymes in the presence of ILs may also offer processing advantages (Wang et al. 2011; Wahlström and Suurnäkki 2015). After the IL pretreatment, the concentration of the IL has to be relatively low due to the instability of enzymes in biomass-dissolving ILs. Therefore, when using enzymes in applications that are performed in aqueous solutions of hydrophilic ILs the *T. maritima* xylanase can be utilised even at 90 °C in reactions that last at least 1 day, conditions that are too extreme for most other xylanases. It was shown earlier with extremophilic *Pyrococcus horikoshii* endoglucanase that in the lignocellulose hydrolysis, temperatures around 100 °C offer clear processing advantages for high solids reactions when compared to reactions at 70–80 °C. In this respect, the ability of TmXYN10B to function in the same temperature range even in the presence of ILs may offer a possibility for building new high temperature process designs in biocatalysis. However, as the present studies were performed with isolated xylan as a substrate, conclusions cannot be drawn yet about whether the use of this extremophilic xylanase in aqueous IL solutions at high temperatures would improve the treatment of lignocellulose. Nevertheless, the high stability of TmXYN10B in ILs at very high temperatures makes it possible to perform such studies in industrial conditions.

## Electronic supplementary material

Below is the link to the electronic supplementary material.

Supplementary material 1 (PDF 267 kb)
