# Supplementary material for: Hyperthermostable Thermotoga maritima xylanase XYN10B shows high activity at high temperatures in the presence of biomass-dissolving hydrophilic ionic liquids
Source: Extremophiles. 2016 May 30;20:515–24. doi: 10.1007/s00792-016-0841-y (PMC4921120; doi:10.1007/s00792-016-0841-y)
Supplement: Supplementary file 1 — Supplementary material 1 (PDF 267 kb) [file 792_2016_841_MOESM1_ESM.pdf]

# Supplemental Data

## Hyperthermostable *Thermotoga maritima* xylanase XYN10B shows high activity at high temperatures in the presence of biomass-dissolving hydrophilic ionic liquids

Tianyi Yu[a], Sasikala Anbarasan[b], Yawei Wang[a], Kübra Telli[b], Aşkin Sevinç Aslan[b], Zhengding Su[c], Yin Zhou[d], Li Zhang[a], Piia Iivonen[b], Sami Havukainen[b], Tero Mentunen[b], Michael Hummel[e], Herbert Sixta[e], Baris Binay[f], Ossi Turunen[b*], Hairong Xiong[a*]

[a] South-central University for Nationalities, College of Life Science, Wuhan, 430074, China.

[b] Aalto University, School of Chemical Technology, Department of Biotechnology and Chemical Technology, 00076 Aalto, Finland.

[c] Hubei University of Technology, Wuhan, 430068, China

[d] Wuhan Sunhy Biology Co., Ltd, Wuhan, 430074, China

[e] Aalto University, School of Chemical Technology, Department of Forest Products Technology, P.O. Box, 16300, 00076 Aalto, Finland

[f] Gebze Technical University, Department of Bioengineering, 41400 Gebze Kocaeli, Turkey

## Inactivation profiles for TmXYN10B

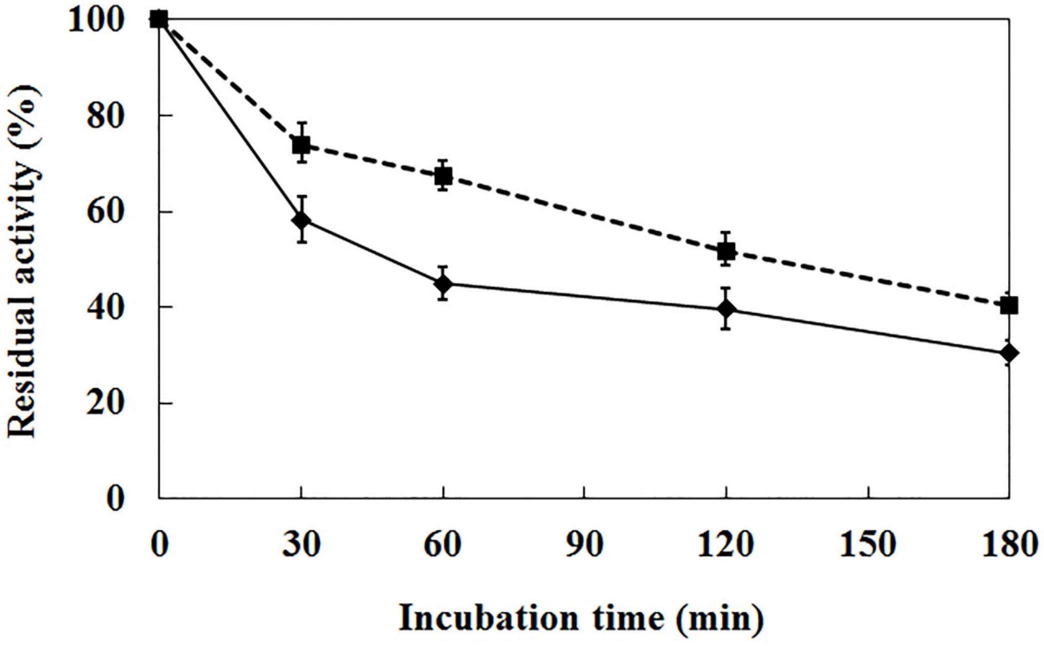


**Fig. S1.** Inactivation profiles of *E. coli*-expressed TmXYN10B (♦) and *P. pastoris*-expressed TmXYN10B (■) at 100° C, pH 5. The half-life of *P. pastoris*-expressed enzyme is 130 min calculated from time range 0-180 min. The initial half-life (0-60 min period) of *E. coli*-expressed is 48 min and for the whole period of 0-180 min the half-life is 78 min.

## Michaelis-Menten hyperbolas

The Michaelis-Menten hyperbolic regression fittings for the kinetic graphs are shown in the Figs. S2A-S2F. The obtained kinetic values are shown in Table 1.

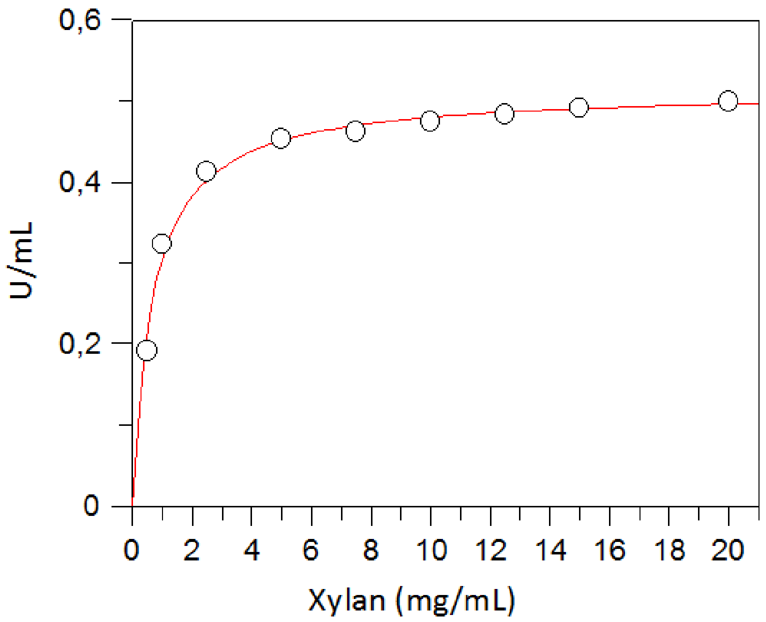


**Fig. S2A. Results from the experiment at 70ºC without ionic liquid (IL).**

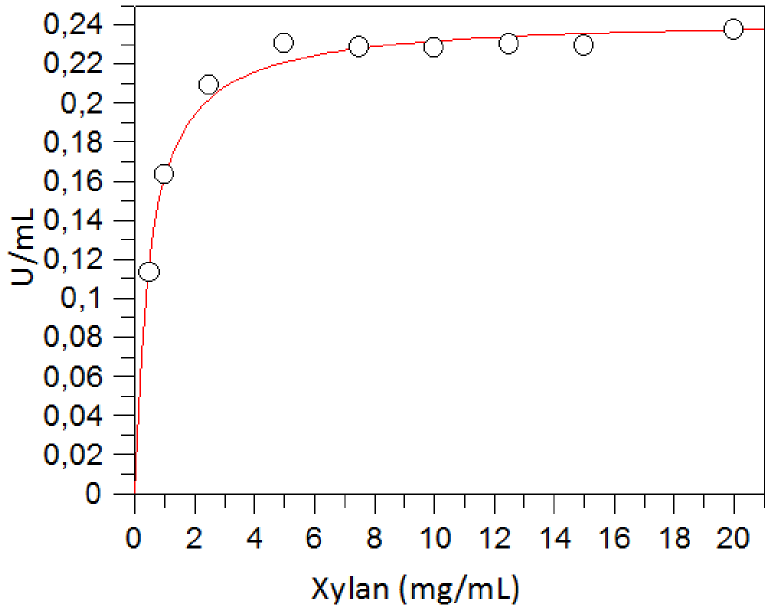


**Fig. S2B. Results from the experiment at 70ºC with [EMIM]OAc.**

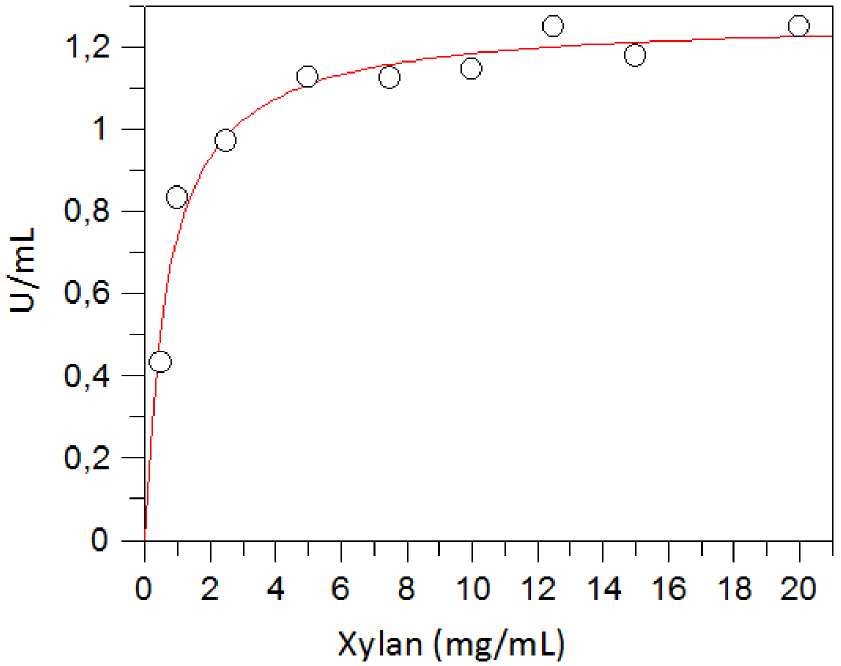


**Fig. S2C. Results from the experiment at 90ºC without IL.**

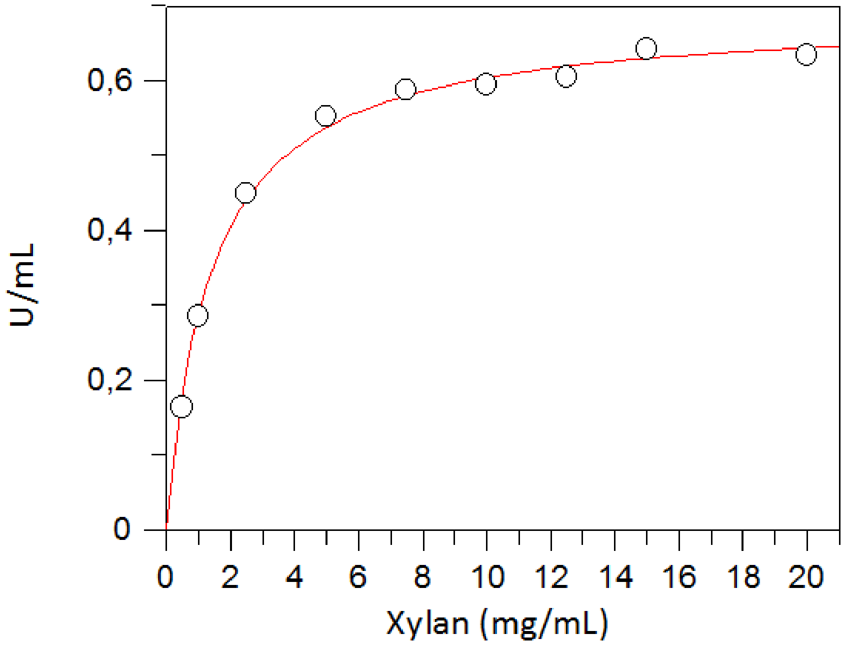


**Fig. S2D. Results from the experiment at 90ºC with [EMIM]OAc.**

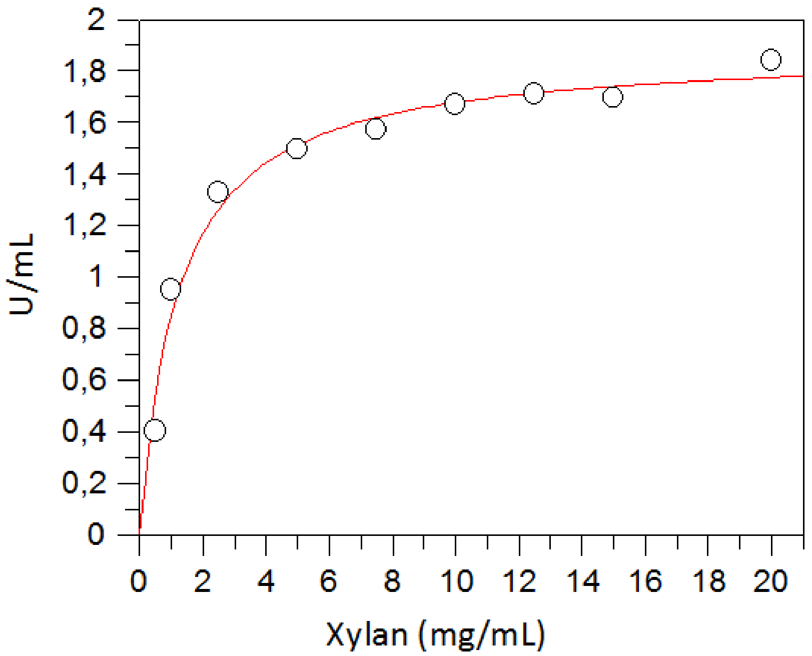


**Fig. S2E. Results from the experiment at 100ºC without IL.**

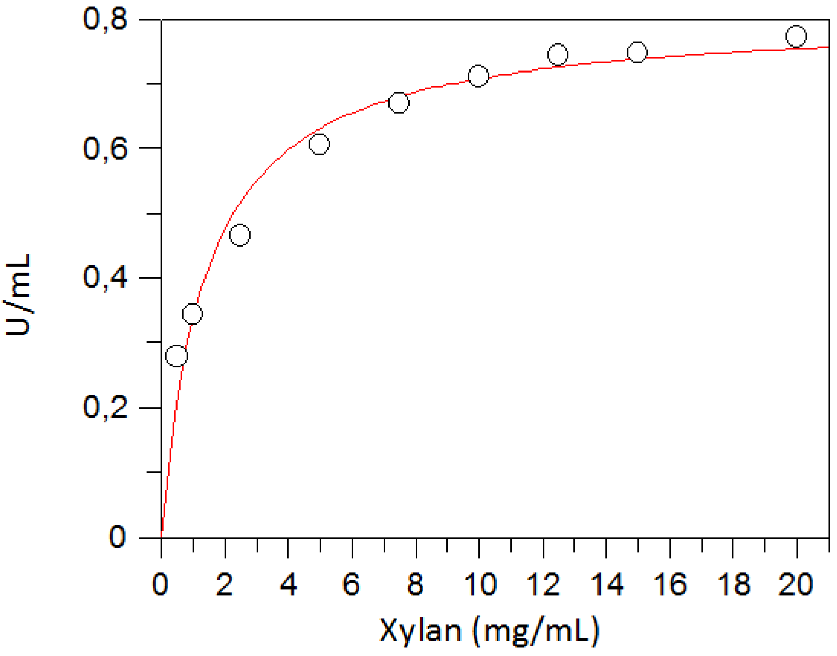


**Fig. S2F. Results from the experiment at 100ºC with [EMIM]OAc.**

## Arrhenius activation energy

Arrhenius activation energies ($E_a$) were calculated from the values of Fig. 4 for TmXYN10B by using the equation ln $k$ = ln $A$ – ($E_a/RT$); $k$ is the rate constant, $A$ is the Arrhenius constant, $R$ is the ideal gas constant, and $T$ is the absolute temperature. A plot of ln $k$ versus 1/$RT$ gives a straight line with slope as $E_a$ (see Fig. S2). The calculated Arrhenius activation energy was: 45.2, 56.9, 66.2, 72.0 kJ/mol for TmXYN10B with 0%, 15%, 25%, 35% [EMIM]OAc, respectively.

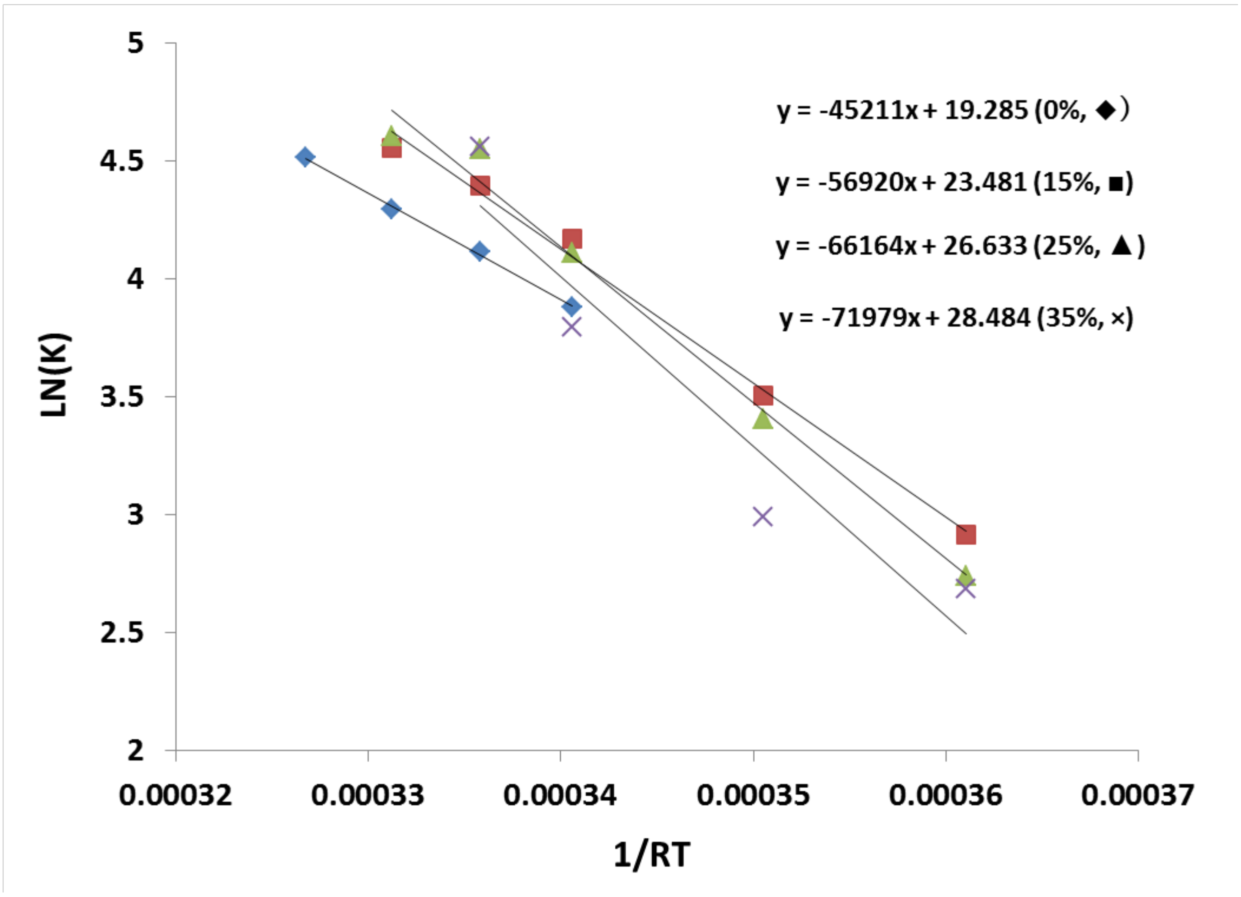


**Fig. S3. Arrhenius plots for temperature-dependent activity of TmXYN10B.** Symbols: without [EMIM]OAc (◆), and with 15% (■), 25% (▲) and 35% (×) [EMIM]OAc.
